# Correction: Impact of non-invasive oxygen reserve index versus standard SpO_2_ monitoring on peripheral oxygen saturation during endotracheal intubation in the intensive care unit: Protocol for the randomized controlled trial NESOI2

**DOI:** 10.1371/journal.pone.0327686

**Published:** 2025-07-02

**Authors:** Hugo Hille, Aurélie Le Thuaut, Pierre Asfar, Quentin Quelven, Emmanuelle Mercier, Anthony Le Meur, Jean-Pierre Quenot, Virginie Lemiale, Grégoire Muller, Martin Cour, Alexis Ferré, Asael Berger, Anaïs Curtiaud, Maxime Touron, Gaetan Plantefeve, Jean-Charles Chakarian, Jean-Damien Ricard, Gwenhael Colin, Arthur Orieux, Patrick Girardie, Mathieu Jozwiak, Manon Rouaud, Camille Juhel, Jean Reignier, Jean-Baptiste Lascarrou, for the CRICS-TRIGGERSEP Network

The 12^th^ author’s name is spelled incorrectly. The correct name is: Asael Berger
