# Supplementary material for: Impact of non-invasive oxygen reserve index versus standard SpO2 monitoring on peripheral oxygen saturation during endotracheal intubation in the intensive care unit: Protocol for the randomized controlled trial NESOI2
Source: PLoS One. 2024 Sep 16;19(9):e0307723. doi: 10.1371/journal.pone.0307723 (PMC11404791; doi:10.1371/journal.pone.0307723)
Supplement: S1 File — (DOCX) [file pone.0307723.s002.docx]

**Protocol NESOI-2**

**Registration No.:** 2023-A00141-41

**Ref:** RC22_0506

**Impact of the non-invasive oxygen-reserve-index versus standard of care on periphereal oxygen saturation during endotracheal intubation in intensive care unit: randomised superiority multi-center 2 arms, open trial**

**Coordinating Investigator:**

Intensive Care

Dr Lascarrou Jean Baptiste

Nantes University Hospital

30 Boulevard Jean Monnet

44093 Nantes Cedex 9

France

Jeanbaptiste.lascarrou@chu-nantes.fr

**Methodology expert:**

Aurélie LE THUAUT

Plateforme de Méthodologie et Biostatistique

Direction de la Recherche et de l’Innovation – CHU de Nantes

Tel: 02 44 76 68 47 / Fax: 02 53 53 62 26

E-mail: aurelie.lethuaut@chu-nantes.fr

**Sponsor:**


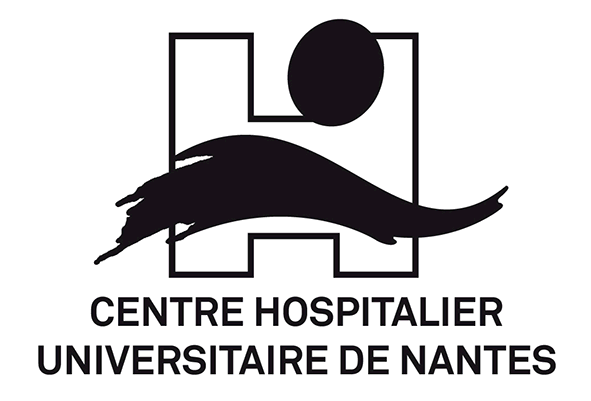
**Nantes University Hospital**
Medical Affairs

and Research Department

5, allée de l’île Gloriette
44 093 Nantes cedex 01 (FRANCE)

Tel: 33 (0)2 53 48 28 35
Fax: 33 (0)2 53 48 28 36

Signature page

**SPONSOR SIGNATURE**

| The sponsor agrees to comply with the laws and regulations on clinical trials for the conduct of the above-mentioned clinical investigation and agrees to abide by all provisions set forth therein. | | |
| --- | --- | --- |
| **Name and capacity of the signatory representative:**  **For the Sponsor and by delegation of the Managing Director, the Director of Research and Innovation** | **Date:** | **Signature:** |

**INVESTIGATOR'S SIGNATURE**

| I have read all the pages of the clinical investigation protocol sponsored by Nantes University Hospital. I confirm that this protocol contains all the information necessary for the conduct of the trial. I agree to conduct the trial according to the protocol and to abide by all provisions set forth therein. I agree to conduct the clinical investigation in compliance with:   - the principles of the "Declaration of Helsinki", - European regulations and/or national laws and regulations relating to clinical trials, - Regulation (EU) 2017/745 on medical devices, - Regulation (EU) 2016/679 of the European Parliament and of the Council of 27 April 2016 on the protection of individuals with regard to the processing of personal data and on the free movement of such data. - the MDCG 2020-10/1,   I also agree for the investigators and other qualified members of my staff to have access to the copies of this protocol and documents concerning the conduct of the study so that they abide by all provisions set forth therein. | | | |
| --- | --- | --- | --- |
| **Coordinating investigator** | **Name:** | **Date:** | **Signature:** |
| **Principal investigator** | **Name and institution:** | **Date:** | **Signature:** |

LIST OF ABBREVIATIONS

| ANSM | Agence Nationale de Sécurité du Médicament et des produits de santé |
| --- | --- |
| MA | Marketing Authorisation |
| CRA | Clinical Research Associate (monitor) |
| GCP | Good Clinical Practice |
| ERB | Ethical Review Board |
| ETI | Endotracheal Intubation |
| CNIL | Commission Nationale de l’Informatique et des Libertés |
| eCRF | Electronic Case Report Form |
| SAE | Serious Adverse Event |
| SAR | Serious Adverse Reaction |
| ICH | International Conference on Harmonization |
| ICU | Intensive Care Unit |
| NIV | Non-Invasive Ventilation |
| PaO2 | Partial-pressure-of-Oxygen |
| RM | Reference Methodology |
| SmPC | Summary of Product Characteristics |
| SOFA | Sequential Organ Failure Assessment |
| SpO2 | Pulse-Oximetry Saturation |
| SUSAR | Suspected Unexpected Serious Adverse Reaction |
| CRT | Clinical Research Technician |
| ORI | Oxygen Reserve Index |
| ETI | Endotracheal intubation |
| NIV | Non-invasive ventilation |
| RCT | Randomized Clinical Trial |

Contents

[Signature page 2](#_Toc146011206)

[LIST OF ABBREVIATIONS 3](#_Toc146011207)

[Contents 4](#_Toc146011208)

[INTRODUCTION 5](#_Toc146011209)

[1. Justification of the study 6](#_Toc146011210)

[1.1. Positioning of the study 6](#_Toc146011211)

[1.2. Benefits and risks for subjects taking part in the study 7](#_Toc146011212)

[1.3. Statement that the research project will be conducted in compliance with the study protocol and with good clinical practice guidelines 9](#_Toc146011213)

[2. Objectives and endpoints 10](#_Toc146011214)

[2.1. Primary objective and endpoint 10](#_Toc146011215)

[2.2. Secondary objectives and endpoints 10](#_Toc146011216)

[3. Study treatments and products 12](#_Toc146011217)

[3.1. Description of the product(s) and mode of administration 12](#_Toc146011218)

[3.2. Authorised and unauthorised treatments in the context of the protocol 12](#_Toc146011219)

[4. Study population 13](#_Toc146011220)

[4.1. Description of the population 13](#_Toc146011221)

[4.2. Inclusion criteria 13](#_Toc146011222)

[4.3. Non-inclusion criteria 13](#_Toc146011223)

[4.4. Recruitment modalities 14](#_Toc146011224)

[5. Study design AND CONDUCT 15](#_Toc146011225)

[5.1. Study schedule 15](#_Toc146011226)

[6. General study methodology 17](#_Toc146011227)

[6.1. Study diagram 17](#_Toc146011228)

[6.2. Description and justification of the treatment plan / method under study 18](#_Toc146011229)

[6.3. Identification of all data sources not included in the medical record 20](#_Toc146011230)

[6.4. Rules for discontinuing subject participation 21](#_Toc146011231)

[7. Data Management AND STATISTICS 22](#_Toc146011232)

[7.1. Data entry and data collection 22](#_Toc146011233)

[7.2. Statistics 23](#_Toc146011234)

[8. Adverse event vigilance and management 27](#_Toc146011235)

[9. Administrative and regulatory aspects 28](#_Toc146011236)

[9.1. Source data and document access rights 28](#_Toc146011237)

[9.2. Trial monitoring 28](#_Toc146011238)

[9.3. Inspection / Audit 29](#_Toc146011239)

[9.4. Ethical considerations 29](#_Toc146011240)

[9.5. Registration/INFORMATION with the competent authorities 30](#_Toc146011241)

[9.6. Amendments to the protocol 30](#_Toc146011242)

[9.7. Study funding and insurance 31](#_Toc146011243)

[9.8. Publication rules 31](#_Toc146011244)

[9.9. Source data archiving 31](#_Toc146011245)

[List of appendices 32](#_Toc146011246)

INTRODUCTION

Intubation is frequently required for patients in the intensive care unit (ICU) but is associated with high morbidity and mortality mainly in emergency procedures and in the presence of severe organ failures. Improving the intubation procedure is a major goal for all ICU physicians worldwide, and improving preoxygenation period may play a relevant role. However, until recently any device which can help physician to evaluate this period when pulse oxygen saturation is upper than 97% were available. Recently Oxygen Reserve Index (ORI) has been introduced by Masimo© as a surrogate of PaO2 in the range between 100 to 250mmHg. We propose to plan a study dedicated to improvement in SpO2 during intubation in ICU by adding ORI monitoring throughout the procedure.

# Justification of the study

## Positioning of the study

Endotracheal intubation (ETI) is a common procedure in ICUs (1). The risk factors in ICU patients result in a high complication rate (up to 50%) (2). Severe complications are severe hypoxaemia (25%), severe hypotension (25%), cardiac arrest (1-3%), and death (0.5-3%) (1,3,4). Risk factors for potentially fatal hypoxaemia are hypoxaemia before ETI and/or difficult ETI (5). Hypoxemia during ETI is associated with cardiac arrest occurrence (3,6) and morbidity.

Predicting hypoxaemia during ETI is challenging (5). Pre-oxygenation is universally recommended to reduce the risk by prolonging the safe apnoea time (7,8). Pulse oximetry saturation (SpO_2_) is monitored in ICU but only detects late-stage hypoxaemia. FeO_2_ is not available for assessing pre-oxygenation efficacy in the ICU. And this technique has limitations in the emergency setting (sensitivity to leaks) and is not available in ICUs (9). Moreover, in critically ill patients, particularly those with acute hypoxemic respiratory failure, FeO_2_ may not be a reliable measure of the effectiveness of preoxygenation: the reduction in functional lung volume leads, on the one hand, to a reduction in FRC (Fonctional Residual Capacity) and, on the other hand, to a shunt that reduces the efficiency of the alveolar-capillary interface (10). The result is that both SpO_2_ and FeO_2_ can be high while PaO_2_ is low. PaO_2_ can be considered the reference standard for evaluating the effectiveness of preoxygenation but unfortunately cannot currently be obtained at the bedside in real-time in clinical practice (9). Non-invasive ventilation (NIV) is currently recommended for patients with severe hypoxaemia but is associated with a higher risk of severe hypotension (11–15). Last, extended duration of preoxygenation did not help to increase quality of its own (16). Thus, at present, the effectiveness of pre-oxygenation cannot be evaluated in ICUs. Predicting the risk of hypoxaemia during ETI and optimising pre-oxygenation are two major therapeutic goals. A reliable tool for assessing the quality of pre-oxygenation is therefore needed.

The oxygen reserve index (ORI) is a new oxygenation-monitoring parameter measured continuously and non-invasively using a specific SpO_2_ sensor (Masimo Inc., Irvine, CA). ORI is a relative indicator of partial-pressure-of-oxygen (PaO_2)_ changes in the moderate hyperoxaemia range (17). ORI recording requires no additional intervention by clinicians in charge of the patient. The ORI is a dimensionless parameter that can range from 0.00 (PaO_2_<100mmHg) to 1.00 (PaO_2_>200mmHg). These were only prospective observational studies conducted on small numbers of patients in the context of planned surgery. Reported time intervals between the ORI decline below 0.4 and desaturation were 30 [20-60] seconds (18,19). We recently reported that, in non-hypoxaemic patients, the median time from an ORI fall below 0.4 and an SpO_2_ fall below 97% during apnoea was 81-s [Q1-Q3: 34-146], which is sufficient to allow preventive interventions. We recently observed than in non-hypoxemic patients, ORI decrease below 0.4 with a 81-s [34-146] median time before the SpO_2_ decrease below 97% during apnea which may allow preventive action. Interestingly, a higher ORI value during preoxygenation was independently protective against hypoxemia (20).

To help determine whether ORI monitoring can minimise hypoxaemia during ETI in critically ill patients, we designed a multicentre randomised controlled trial (RCT) to determine whether ORI monitoring is superior over standard care in preventing hypoxaemia.

## Benefits and risks for subjects taking part in the study

### Benefits

#### Individual benefit

If our hypothesis is verified, compared to usual care, use of ORI during preoxygenation will lead to increase in oxygenation parameters with higher safety for patient.

#### Collective benefit

The expected benefits of RCT for patients is better pre-oxygenation with less subsequent hypoxaemia when ETI is required in the ICU. ORI monitoring is expected to result in earlier recognition of impending hypoxaemia (when combined with other changes in the ETI procedure), thus improving the safety of ETI in the ICU. These benefits will be relevant to the very large number of ICU patients who undergo ETI worldwide.

### Risks

#### Individual risk

- Physical risks and constraints

Risks for patients included in the study are negligible. Previous studies on use of ORI during intubation in intensive care unit showed a good tolerance profile (20).

- Disease-related risks

Patients eligible for study inclusion will have a critical illness associated with a high risk of complications including nosocomial infections, organ failure, metabolic disorders, and subsequent death. Care given to the study patients will be appropriate for their health condition, as used routinely in the participating ICUs and as decided by the bedside physicians and ICU teams. Thus, this research will not increase disease-related risks.

#### Collective risk

The NESOI-2 study is not associated with any collective risk. This study will not generate additional costs for the participating centres.

### Benefit / risk balance

Given the characteristics of the research project, the chief investigator considers that this research has minimal risks and constraints, based on the following arguments:

- Current practice for peri-intubation procedures in intensive care unit are heterogeneous (21);
- Few adverse event occurred during observational study dedicated to evaluation of ORI monitoring (20);
- ORI monitoring has a CE marking approval;

In conclusion, given the potential benefits previously stated in point 1.2.1 and the minimal risks and constraints associated with the research, the risk/benefit ratio is extremely favourable.

## Statement that the research project will be conducted in compliance with the study protocol and with good clinical practice guidelines

The investigator undertakes to ensure that this research is conducted:

- - In compliance with the study protocol, and
  - In compliance with current French and international good clinical practice guidelines.

**The bibliographical references are appended to the document.**

# Objectives and endpoints

## Primary objective and endpoint

### Primary objective

The primary objective is to determine whether ORI monitoring increases the lowest oxygen saturation level during the interval between the first laryngoscopy (defined as introduction of the laryngoscope into the mouth) and the end of the second minute after successful ETI.

### Primary endpoint

Lowest oxygen saturation, measured by Masimo Rad7 oximeter, during the interval between the first laryngoscopy (defined as introduction of the laryngoscope into the mouth) and the end of the second minute after successful ETI (22).

## Secondary objectives and endpoints

### Secondary objective(s)

- To determine whether ORI monitoring increases the lowest oxygen saturation level according to local oximeter.

- To determine whether ORI monitoring increases the lowest oxygen saturation level according to subgroups.

- To assess safety of ORI monitoring.

- To assess efficacy of ORI monitoring.

### Secondary endpoint(s)

- Sensitivity analysis of primary endpoint
  - Lowest oxygen saturation, measured by local oximeter, during the interval between the first laryngoscopy (defined as introduction of the laryngoscope into the mouth) and the end of the second minute after successful ETI.
- Sub-group analysis of primary endpoint
  - Lowest oxygen saturation in pre-defined subgroups
    - Body mass index (<30 or ≥30)
    - Reason for ETI: hypoxaemia/other
    - Shock/No shock at inclusion
    - Difficult intubation (yes or no)
    - ORI threshold (<0.6 or ≥0.6)

- Safety outcome

- - Occurrence of at least one severe life-threatening complication: death, cardiac arrest, arterial systolic pressure <90 mmHg, and/or SpO_2_<80% and occurrence of each one.

- Efficacy outcome

- - ICU mortality
  - 28-day mortality
  - ICU and hospital stay length
  - Mean cognitive score at day 28 between two groups (modified Telephone Interview for Cognitive Status (F-TICS-m) (23)

# Study treatments and products

## Description of the product(s) and mode of administration

### Experimental product(s)

The oxygen reserve index (ORI) is a new oxygenation monitoring parameter measured continuously and noninvasively. It can be obtained from specific SpO2 sensor manufactured by Masimo (Masimo Inc., Irvine, CA). It is a relative indicator of partial pressure of oxygen (PaO_2)_ changes in the moderate hyperoxemia range. It did not require additional intervention from doctor or nurse in charge of the patient. The ORI is a dimensionless parameter that can range from 0.00 (PaO2<100mmHg) to 1.00 (PaO2>200mmHg). It is measured by a multi-wavelength pulse co-oximeter placed on the tip of a finger (Rainbow SET, Masimo Inc., Irvine, CA) (17). The device analyzes variations in the pulsatile blood absorption of incident light at both the arterial and venous levels. It is a relative indicator of PaO_2_ changes in the moderate hyperoxemia range. When pure oxygen is administered, SaO_2_ reaches 100% when PaO_2_ reaches 100 mmHg. Beyond that, PaO_2_ continues to increase and both SaO_2_ and SpO_2_ remain at 100%, whereas the ORI increases non-linearly from 0.00 (PaO_2_~100 mm Hg) to 1.00 (PaO_2_~200 mmHg). Dedicated sensors will be provided by Masimo for study purpose.

## Authorised and unauthorised treatments in the context of the protocol

### Authorised treatments

All medications whose use in standard practice in ICU patients will be authorised for use according to the specific needs of each patient.

### Unauthorised treatments

No treatment used according to its marketing authorisation in contra-indicated in this trial.

# Study population

## Description of the population

The trial population will be adults (18 years of age or older) who need endotracheal intubation while hospitalized in ICU. Patients will be eligible for enrolment if they meet all the following inclusion criteria and none of the non-inclusion criteria.

Patients participating in intubation research with an oxygenation endpoint will not be eligible for inclusion in the NESOI-2 protocol.

## Inclusion criteria

- ICU admission and need for ETI to allow mechanical ventilation
- Need for supplemental oxygen (via any device and at any flow rate) to obtain SpO_2_>97%
- Patient or next of kin informed about the study and having consented to participation of the patient in the study (patients with coma are unable to consent). If patient is no competent and no next of kin can be contacted during screening for the study, trial inclusion will be completed as an emergency procedure by the ICU physician, in compliance with French law
- Patients affiliated to a social security system

## Non-inclusion criteria

- Fiberoptic intubation required according to physician in charge
- Contra-indications to laryngoscopy (e.g., unstable spinal lesion)
- Insufficient time to include and randomise the patient (e.g., because of cardiac arrest)
- Age <18 years
- Currently pregnant or breastfeeding
- Correctional facility inmate
- Under guardianship, curatorship or under protection of justice

## Recruitment modalities

The recruitment will be done within the ICU departments of the centers participating in the study. It is planned to include 950 patients admitted to the intensive care unit and justifying an orotracheal intubation procedure, after reading the information letter and collecting the consent of the patient or the trusted person, or carrying out the emergency inclusion procedure. In the last two cases, the patient's consent will be collected posteriori after reading the information letter. Patient will be followed until day 28 or death.

# Study design AND CONDUCT

## Study schedule

Consecutive ICU patients requiring ETI will be randomly allocated to ETI with or without ORI monitoring before beginning of preoxygenation, with stratification by centre, expert or nonexpert intubator status (experts: ≥5 years’ ICU experience, or ≥1 year ICU experience plus ≥2 years’ anaesthesiology training) (24), and NIV or others devices for preoxygenation. Two intubators including at least one expert will be present throughout ETI. At least one of the intubators will be trained in ORI monitoring, i.e., will have attended an education session and used the device twice.

Randomisation will be via an electronic case-report form, using a balanced scheme with stratification as described above.

Informed consent will be obtained from the patient or next of kin.

Patients for whom no family member is available to receive information and consent to participate in the study within a time frame compatible with the study design will be included in the study after completion of an emergency consent form by the patient's attending physician. Informed consent will be sought from the patient upon regaining decision-making capacity.

Data collected:

- Patient characteristics: demographics, Simplified Acute Physiologic Score II, activity level (Knaus), Charlson comorbidity index, ICU admission diagnosis, reason for ETI, Sequential Organ Failure Assessment [SOFA] score, vital signs at enrolment, criteria for difficult face-mask ventilation, criteria for difficult intubation, and MACOCHA score
- Before, during ETI, and 2 minutes after successful ETI: ORI, Mallampati score, pre-oxygenation methods and duration, type and dosage of sedatives and neuromuscular blockers, Cormack-Lehane grade, glottic opening score, number of ETI attempts before success (each introduction of the laryngoscope into the mouth is a separate laryngoscopy attempt), with details: manoeuvres during laryngoscopy, reason for ETI failure, difficult ETI , duration of ETI , need for face-mask ventilation after first ETI attempt, need for gum elastic bougie, and types of life-threatening and other complications. Pulse oximetry will be recorded throughout the ETI procedure.
- Day 1 to 7: SOFA score
- Day 28: Mortality, ICU stay length, hospital stay length, cognitive score (F-TICS-m)

**STUDY SCHEDULE**

| **Activities** | D0  (Inclusion visit) | D1 to D28 | D28 |
| --- | --- | --- | --- |
| Participant information | X |  |  |
| Consent | X |  |  |
| Randomization | X |  |  |
| Previous medical history | X |  |  |
| Clinical examination | X | X |  |
| Efficacy and/or safety assessment | X | X |  |
| Para-clinical examinations | X |  |  |
| Compliance | X |  |  |
| Adverse events |  | X | X |
| Phone contacts |  |  | X |

# General study methodology

The study presents the following characteristics:

- Study of preoxygenation during intubation in intensive care,
- Multi-centre national study,
- Controlled, superiority, 2 arms,
- Randomised stratified,
- Open label trial, with unified outcome assessmentParallel group study.

## Study diagram


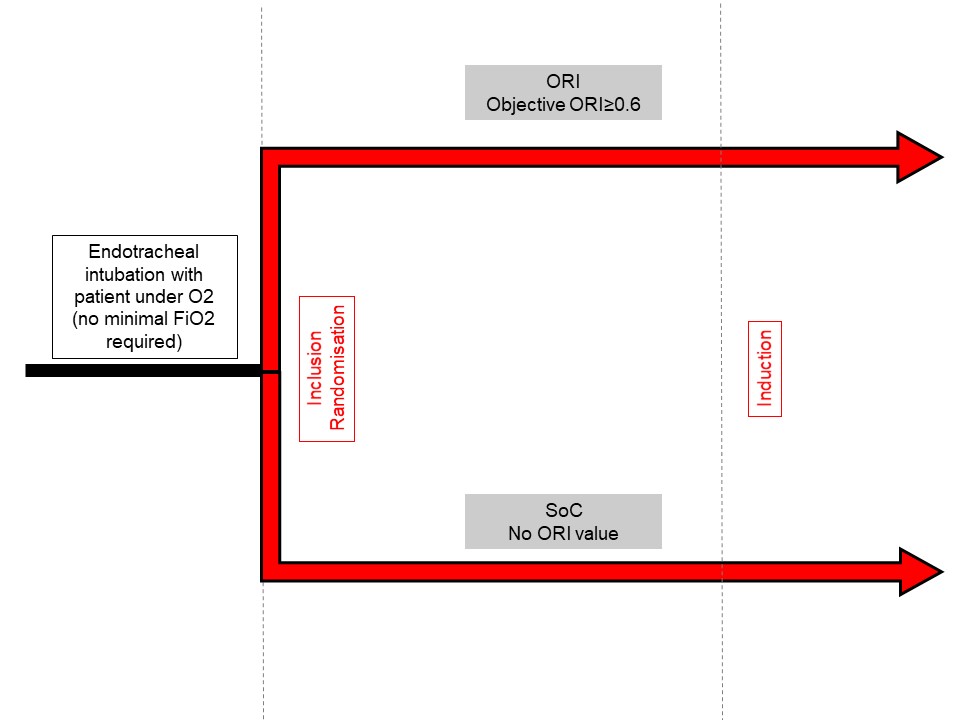


SoC: Standard of Care

## Description and justification of the treatment plan / method under study

### Treatment plan

Monitoring of the SpO2 will be determined at the beginning of the intubation procedure continuously by 2 sensors (local sensor, and Masimo Rad 7) for all patients. All values will be entered into an eCRF. Each sensor will be placed on index, middle and ring fingers of each hand.

Evaluation of difficult intubation and difficult ventilation will be performed in accordance to factors previously validated: This evaluation will be captured in the electronic case report form (body mass index, mouth opening, thyro-mental distance, Mallampati score, snorer, toothless, sleep apnea) according to De Jong *et al* (25).

ETI is performed in both groups according to the protocol outlined below.

(a) Pre-oxygenation is achieved using the device chosen by the doctor in charge of the patient:

- Bag valve mask delivering oxygen at a minimum flow of 60 L/minute for at least 3 minutes (26–28);
- Non-rebreathing (high-concentration) mask delivering oxygen at a minimum flow of 60 L/ minute for at least 3 minutes (28,29);
- Ventilator in NIV mode providing 100% FiO_2_ for at least 3 minutes (12);
- High-flow nasal oxygen device (e.g., Optiflow^®^) delivering oxygen at a minimum flow of 60 L/minute, with 100% FiO_2_, for at least 3 minutes (30,31).

Choice of preoxygenation device will be at physician discretion but Non Invasive Ventilation will be advised in case of oxygen flow > 8l/min to obtain SpO2≥95%. Additionally, apneic ventilation will be also advised (22).

Preoxygenation will be delivered according to protocol defined with each local primary investigators. Cut-off of 0.6 for ORI objective at the end of the preoxygenation was adopted according to re-analysis of previous data (personal data, not published).


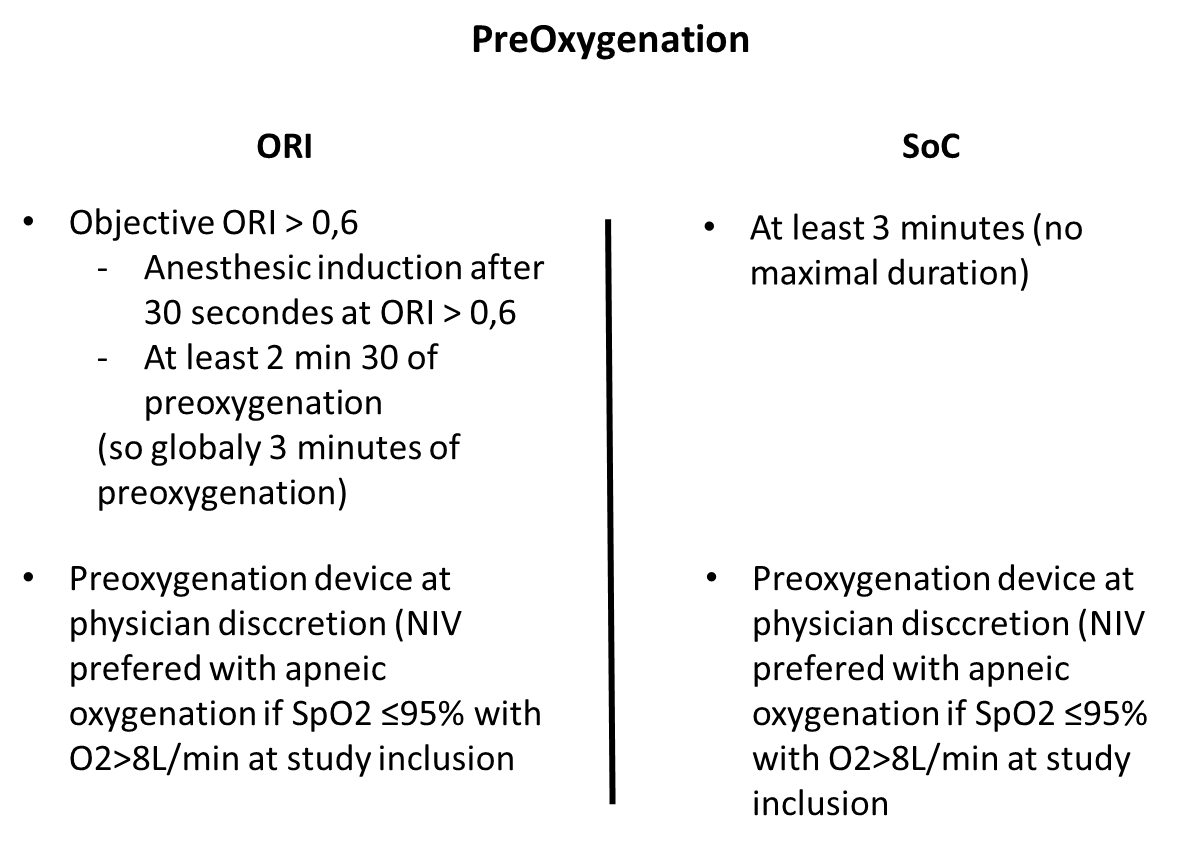


(b) Anaesthesia is then induced by injecting a hypnotic agent and a neuromuscular blocking agent. The type and dosage of these drugs is at the discretion of the doctor performing the intubation. Nevertheless, in agreement with international (7) and French guidelines (8), the following two principles are applied:

- The preferred neuromuscular blocking agent in the absence of contra-indications (e.g., hyperkalaemia, burn injury more than 24 hours earlier, spinal lesion, or allergy) is succinylcholine in a dosage of 1 mg/kg. The alternative is rocuronium 1 mg/kg, provided the antidote sugammadex (16 mg/kg) is available.
- If possible, the hypnotic agent is either hypnomidate 0.2-0.3 mg/kg or ketamine 1-2 mg/kg.

(c) Laryngoscopy is performed using the device choose by physician in charge. The size of endotracheal tube and the size of the Macintosh laryngoscope were chosen by the physician at bedside.

(d) ETI is then performed. The cuff of the endotracheal tube is inflated and the tube is connected to the ventilator. If this first ETI attempt fails, the doctor chooses between a second laryngoscopy and an alternative ETI technique. Alternative ETI technique will be chosen at physician discretion and in accordance to French guidelines (7). Each introduction of the laryngoscope into the oral cavity of the patient is considered a separate laryngoscopy attempt. Use of Sellick’s manoeuver [pressure applied to the cricoid cartilage] during ETI is at the discretion of the doctor in charge of the patient and is recorded in the electronic case-report form [eCRF]. Intra-tracheal tube position is confirmed by analysing the capnography curve over four or more breathing cycles. Total ETI duration is defined as the time from anaesthesia induction initiation to observation of the first inflection on the expired capnography curve (confirming intra-tracheal tube position). All items of electronic case report form were analysis on real time by a third person not in charge of patient’s care.

During intubation procedure, blinded continuous monitoring of SpO2 and ORI (for control group) will be performed by appropriate monitor (Masimo Rad7) with data extraction at regular intervals. Oxygen saturation will be tracked during ETI by research nurse or assistant nurse dedicated to fill the eCRF. ORI evolution will not be provided to investigators in the standard of care group. In the control group, local continuous monitoring of SpO2 will be performed with local pulse oximeter device.

## Identification of all data sources not included in the medical record

Clinical, laboratory and background data will be collected at the time of enrolment, during the ICU-stay, at ICU-discharge, at hospital-discharge, and at follow-up. Data will be obtained from hospital records, relatives, and will be entered into a web-based electronic case record form (eCRF) by site personnel. The site investigator must sign all eCRFs before trial completion to verify that the recorded data is correct and complete. Data from the web-based forms will be migrated to a trial database, which will be handled by the coordinating team. Data not obtainable will be registered as missing and measures to obtain data should not delay intervention or concomitant treatment (i.e. central line not in place at the time of data collection).

Collected data will include:

- Baseline: Inclusion and non-inclusion criteria; demographics; significant comorbid diseases
- Data on hospital admission: temperature on admission; neurological assessment; cause of arrest and diagnostic work-up (i.e. EKG, echocardiography …); Sequential Organ Failure Assessment (SOFA) score; presence of shock on admission; administered therapies
- In the ICU: characteristics of intubation process (drugs, duration)
- Daily during the ICU stay: SOFA score; adverse events
- Outcome and therapies received at ICU discharge
- Outcome and cognitive function at 28 days after randomization

## Rules for discontinuing subject participation

### Patient withdrawal from the study

The patients or next of kin may ask to withdraw from the study at any time and for any reason. According to French law, in the event of secondary withdrawal of consent to participation in the research project, the data collected from study inclusion to withdrawal of the patient will be used. Data collection will be stopped in compliance with the wishes of the patient or next of kin. Withdrawal of a patient from the study will have no impact on the standard care delivered to the patient for the condition that prompted the hospital admission. All investigations and treatment decisions will be at the discretion of the physician in charge of the patient.

Should a patient be lost to follow-up, all data for that patient collected since study inclusion will be used, and the investigator will make every effort to contact the patient.

If a patient included in the emergency procedure died before the collection of his or her consent to continue and no next-of-kin consent could be collected, then his or her data will not be retained and that patient will be excluded from the analysis.

### Discontinuation of all part of the study

The study will be completed after collection of the day 28 data for the patient included last in the study. Given that this research investigates standard practice and has minimal risks and constraints, the only pre-defined study discontinuation criterion is a scientific publication resulting in a consensus that challenges the primary study hypothesis or the method used to optimize oxygenation during intubation for patient in intensive care.

# Data Management AND STATISTICS

## Data entry and data collection

All the study data are collected routinely in ICUs for the management of comatose mechanically ventilated patients:

- Demographics and clinical data
- Treatments given: daily until hospital discharge
- Laboratory data
- Nosocomial infections
- Invasive devices

### Data entry, processing and circulation

Data collection for each person participating in the research is realized with an electronic case report form (eCRF).

Each person responsible for the filling of the eCRF (investigator, ARC ...):

• will have to be identified in the table of delegations of responsibilities of each center (see investigator’s file).

• Will have a “user” account with specific computer rights linked to his role (right to enter or modify a data, right to lock, monitor or sign a page of eCRF ...)

Entering, viewing or modifying data will only be possible via the eCRF pages (input masks), on <https://nantes-lrsy.hugo-online.fr/CSonline>.

The data will be stored directly from the eCRF into the database hosted on a dedicated server, with controlled access (account/password) according to the user role. Any addition, modification or deletion of data will be recorded in a non-editable electronic file (the audit trail)

### Participant identification

The principal investigator and all co-investigators undertake to keep the identities of the patients who participate in the study confidential, by assigning a coded identifier to each patient.

This patient identifier will be present on all eCRF pages and all attached documents (reports of imaging studies, laboratory tests, health economics analysis, etc.). It will be the only information allowing retrospective connection of the data to the patient.

The coded identifier will be the first letter of the patient’s first name, the first letter of the patient’s surname, the birth year of the patient, and the study inclusion number, in that order.

## Statistics

### Description of planned statistical methods, including planned intermediate analysis schedule

Statistical analyses will be performed by a biostatistician from the methodology and biostatistics platform using SAS or R or Stata.

A data review will be carried out before the freezing of clinical trial data bases, and statistical analyses, with the main stakeholders in the study (investigator, CRA, data manager and biostatistician…). The objective of this review will be to take stock of any difficulties encountered during the study, and to classify the different deviations in order to define the analysis populations.

The statistical analysis will be conducted according to a pre-defined plan.

**Description of the groups at baseline**

Baseline characteristics will be studied using descriptive statistics. No statistical tests will be performed.

**Analysis of primary endpoint**

Lowest oxygen saturation, measured by Masimo Rad7 oximeter, during the interval between the first laryngoscopy (defined as introduction of the laryngoscope into the mouth) and the end of the second minute after successful ETI (32) will be compared between groups using linear mixed-effect regression model to take into account stratification variable (centre as random effect, intubator status (expert or non-expert) and preoxygenation device as fixed effect).

**Sensitivity analysis of primary endpoint**

The same analysis will be realized using the lowest oxygen saturation measured by local oximeter.

**Analysis of primary endpoint according to subgroup**

Primary endpoint will be analyzed according to pre-defined subgroups variables:

- - - Body mass index (<30 or ≥30)
    - Reason for ETI: hypoxaemia/other
    - Shock/No shock at inclusion
    - Difficult intubation (yes or no)
    - ORI threshold (<0.6 or ≥0.6)

**Analysis of secondary endpoints**

All analyses will be adjusted on stratification criterion.

- Occurrence of at least one severe life-threatening complication: death, cardiac arrest, arterial systolic pressure <90 mmHg, and/or SpO_2_<80% and occurrence of each one using logistic mixed-effect regression model.

- The rates of death in ICU and at 28 days will be calculated via Kaplan-Meier plots and compared between groups using Cox regression models.

- ICU and hospital length of stay will be compared using Fine and Gray model to take into account death as competing risk.

- Cognitive score evaluated by F-TICS-m will be compared between groups using linear mixed effect regression model. Patients dead at day 28 will be not included on this analysis.

### Statistical justification of the number of inclusions

Assuming a standard deviation of 10 at the lowest oxygen saturation in control group (33), no missing data, and cross-over for 5% of patients, with the two-sided alpha risk set at 0.05, including 950 patients will provide 85% power for detecting an absolute 2-percentage points between-group difference in lowest oxygen saturation (3% difference in FLORALI2 trial (12) and 96 vs 93% in PREVENT study (22)).

### Envisaged degree of statistical significance

The statistical tests will be performed using a significance threshold of 5%.

### Consideration method for missing, unused or invalid data

In case of missing data on primary endpoint, multiple imputations will be realized. No imputation will be realized on secondary endpoints.

### Management of changes made to the initial analytical strategy

No changes to the statistical analysis plan are planned.

### Choice of subjects to be included in analysis

Modified Intention-to-treat (mITT) analyses will be performed. This population will exclude patient with no legal requirement.

Per protocol analyses will also be performed and will exclude patient with failure of ORI capture, failure of ORI recording or ORI<0.6 at the end of the procedure.

### Randomisation

Randomization will be stratified by centre and expert or non-expert intubator status (experts: ≥5 years’ ICU experience, or ≥ 1 year ICU experience plus ≥2 years’ anaesthesiology training), preoxygenation devices (NIV, others).

It will be performed according to a 1:1 ratio and will be carried out in blocks.

The randomization will be performed under Ennov Clinical by connecting to the website: https://nantes-lrsy.hugo-online.fr/CSonline. The connection will be done with a login, a password and a study number, delivered by the data manager of the Research Unit of the CHU Nantes. The following information must be filled in:

- First initial of the name,

- First initial of the first name,

- Month and year of birth,

- Compliance with the inclusion and non-inclusion criteria (yes/no).

Randomization will be performed by the investigator after confirmation of the possibility of inclusion in the study. The inclusion number will be assigned automatically during randomization. An e-mail confirmation will be sent to the person who performed the randomization and to all the persons concerned.

The randomization list will be made by the statistician in charge of the study. An explanatory guide to randomization will be available online under Ennov Clinical.

# Adverse event vigilance and management

As the protocol is with minimal risks and constraints, no adverse event associated with this study is expected.

The onset of an Adverse Reaction associated with patient care in the course of this protocol shall be reported in the suitable vigilance system (pharmacovigilance, biovigilance, haemovigilance, medical device vigilance, etc.).

# Administrative and regulatory aspects

## Source data and document access rights

Each person's medical data shall only be provided to the sponsor or any person duly authorised by the sponsor, and, where applicable, to authorised health authorities, in confidential conditions.

The sponsor and the supervisory authorities may request direct access to medical records for the purposes of verification of the procedures and/or data in respect of the clinical trial, within the limits authorised by the legislation and regulations.

The data compiled during the trial may be processed electronically in compliance with CNIL requirements.

## Trial monitoring

Monitoring shall be carried out by the Research Division Promotion Department. A Clinical Research Associate (CRA) shall visit each site (investigator and dispensary) regularly to conduct quality control on the data reported in the case report forms.

The protocol has been classified according to the estimated level of risk for the person taking part in the study. It shall be monitored as follows: Risk B: foreseeable risk similar to that of standard care

The on-site monitoring visits shall be organised after making arrangements with the investigator. The CRAs should be able to consult on each site:

- the enrolled persons' data compilation records,

- the patients' medical and nursing files,

- the investigator file,

- the distribution and storage site of the experimental product

## Inspection / Audit

Within the scope of this study, an inspection or audit may be conducted. The sponsor and/or participating centres should be able to provide inspectors or auditors with access to the data.

## Ethical considerations

### Written informed consent

The investigator agrees to provide the subject with clear and precise information about the protocol and request from him/her a written consent. The investigator shall give the subject a copy of the information form and consent form. The subject can only be enrolled in the study after reading the information form and giving his/her written consent form, after taking time to reflect on the matter.

The investigator shall also sign and date the consent form. Both documents should be issued at least in duplicate hard copy format so that the person and the investigator can each keep a copy. The investigator's original shall be placed in the investigator file. If the consent form is signed in duplicate, the investigator keeps the original and gives the copy to the subject.

For patients unable to give consent at the time of inclusion due to impaired consciousness, their next of kin will be given full and honest information, delivered in easily understandable terms, about the study objectives and their right to refuse their relative's participation in the study or to withdraw their relative from the study at any time. Information and informed consent documents created specifically for the study will be given to the next of kin. The participant will be informed as soon as possible and consent will be sought for possible continuation of the research if capacity to consent is restored.

The investigator will also sign and date the consent form and give a copy of the information and consent forms to the patient or next of kin. At least two hard copies of both documents should be issued so that the patient or next of kin and the investigator can each retain a copy.

### Emergency consent form collection

If the family member is not present, it will be derogated to the consent collection obligation at the time of inclusion. This request will be appreciated by the Ethics Committee (EC). Patients for whom no relative is available to receive information and consent to study participation within a timeframe compatible with the study design, will be included in the study after a statement from the physician in charge of the patient confirming that the patient is not available to receive the information, that the situation is an emergency and that no family member is present. Informed consent will be sought from the relative as soon as possible and then from the patient as soon as he or she recovers decision-making competency.

### Ethical Review Board

The sponsor undertakes to submit the draft study to the Ethical Review Board (ERB) for prior approval.

## Registration/INFORMATION with the competent authorities

This protocol shall be the subject of an ANSM information.

## Amendments to the protocol

Requests for substantial modifications should be addressed by the sponsor for notification to ANSM and/or the Ethical Review Board concerned in compliance with the applicable law and its implementing decrees.

The amended protocol should be a dated updated version.

The information and consent forms should be amended if required.

## Study funding and insurance

The sponsor shall fund the study and take out an insurance policy covering the financial consequences of its civil liability in compliance with the regulations.

## Publication rules

Authorship will be granted using the Vancouver definitions and depending on personal involvement and fulfilment of the author’s respective roles. The main publication will report the primary and secondary outcomes. A copy of the publication shall be delivered to Nantes University Hospital, the study sponsor, which shall necessarily be cited. The following statement will also be made in all communications and scientific reports relevant to this study: “This study was supported by a grant from the French Ministry of Health (PHRC-21-0202).

## Source data archiving

The investigator should archive all study data for at least 15 years after the end of the study. At the end of the study, the investigator shall also receive a copy of the data for each person in the investigator's centre sent by the sponsor.

List of appendices

- **Appendix 1: Investigator List**
- **Appendix 2: Summary of Protocol**
- **Appendix 3: Bibliographic references**
- **Appendix 4: Emergency situation attestation form**
- **Appendix 5: Patient information form**
- **Appendix 5bis: Relative consent form**
- **Appendix 6: Relative information form**
- **Appendix 6bis: Relative consent form**
- **Appendix 7: Patient information form, after emergency situation**
- **Appendix 7bis: Patient consent form after emergency situation**
- **Appendix 8: Relative information form, after emergency situation**
- **Appendix 8bis: Relative consent form, after emergency situation**

Appendix 1: Investigator list

Appendix 2: SUMMARY of protocol

| **Title of study** | **Impact of the non-invasive oxygen-reserve-index versus standard of care on periphereal oxygen saturation during endotracheal intubation in intensive care unit: randomised superiority multi-center 2 arms, open trial** |
| --- | --- |
| **Keywords** | Intubation, hypoxemia, intensive care unit, acute respiratory failure |
| **Sponsor of study** | **NANTES University Hospital** |
| **Coordinator**  **(if multi-centre)** | Dr LASCARROU Jean Baptiste  Médecine Intensive Réanimation  Nantes University Hospital |
| **Number of centres**  **envisaged** | 20 National study |
| **Study duration** | - Total duration:  13 months - Enrolment period: 12 months - Participant treatment period: 1 hour - Participant follow-up period: 28 days |
| **Study type and design** | - Therapeutics - Multi-centre - Controlled, superiority - Randomised - Open label trial, with unified outcome assessment - Prospective |
| **Projected number of cases** | 950 |
| **Study objectives** | Primary objective: The primary objective is to determine whether ORI monitoring increases the lowest oxygen saturation level during the interval between the first laryngoscopy (defined as introduction of the laryngoscope into the mouth) and the end of the second minute after successful ETI.  Secondary objective(s):  - To determine whether ORI monitoring increases the lowest oxygen saturation level according to local oximeter.  - To determine whether ORI monitoring increases the lowest oxygen saturation level according to subgroups.  - To assess safety of ORI monitoring.  - To assess efficacy of ORI monitoring |
| **Study endpoint(s)** | Primary endpoint: lowest oxygen saturation measured by Masimo Rad7 oximeter, during the interval between the first laryngoscopy (defined as introduction of the laryngoscope into the mouth) and the end of the second minute after successful ETI.  Secondary endpoint (s):   - Sensitivity analysis of primary endpoint : - Lowest oxygen saturation, measured by local oximeter, during the interval between the first laryngoscopy. - Sub-group analysis of primary endpoint - Lowest oxygen saturation in pre-defined subgroups   - Body mass index (<30 or ≥30)   - Reason for ETI: hypoxaemia/other   - Shock/No shock at inclusion   - Difficult intubation (yes or no)   ORI threshold (<0.6 or ≥0.6) Safety outcome:   - Occurrence of at least one severe life-threatening complication: death, cardiac arrest, arterial systolic pressure <90 mmHg, and/or SpO_2_<80% and occurrence of each one.   - Efficacy outcome   - ICU mortality - 28-day mortalityICU and hospital stay length |
| **Treatment, interventional procedure under study** | ORI monitoring during preoxygenation of patients admitted in intensive care unit who required oxygen supplementation |
| **Comparator (if applicable)** | Standard of care (3 minutes of preoxygenation with device at physician discretion) |
| **Main selection, inclusion, non-inclusion and exclusion criteria** | Inclusion criteria   - ICU admission and need for ETI to allow mechanical ventilation - Need for supplemental oxygen (via any device and at any flow rate) to obtain SpO_2_>97% - Patient or next of kin informed about the study and having consented to participation of the patient in the study (patients with coma are unable to consent). If patient is no competent and no next of kin can be contacted during screening for the study, trial inclusion will be completed as an emergency procedure by the ICU physician, in compliance with French law - Patients affiliated to a social security system   Non-inclusion criteria   - Fiberoptic intubation required according to physician in charge - contra-indications to laryngoscopy (e.g., unstable spinal lesion) - insufficient time to include and randomise the patient (e.g., because of cardiac arrest) - age <18 years - currently pregnant or breastfeeding - correctional facility inmate - under guardianship, curatorship or under protection of justice |
| **Schedule of the various visits and examinations** | \| **Activities** \| D0  (Inclusion visit) \| D1 to D28 \| D28 \| \| --- \| --- \| --- \| --- \| \| Participant information \| X \|  \|  \| \| Consent \| X \|  \|  \| \| Randomization \| X \|  \|  \| \| Previous medical history \| X \|  \|  \| \| Clinical examination \| X \| X \|  \| \| Efficacy and/or safety assessment \| X \| X \|  \| \| Para-clinical examinations \| X \|  \|  \| \| Compliance \| X \|  \|  \| \| Adverse events \|  \| X \| X \| \| Phone contacts \|  \|  \| X \| |
| **Statistical analysis** | Statistical analyses will be performed by a biostatistician from the methodology and biostatistics platform using SAS or R or Stata.  A data review will be carried out before the freezing of clinical trial data bases, and statistical analyses, with the main stakeholders in the study (investigator, CRA, data manager and biostatistician…). The objective of this review will be to take stock of any difficulties encountered during the study, and to classify the different deviations in order to define the analysis populations.  **Description of the groups at baseline**  Baseline characteristics will be studied using descriptive statistics. No statistical tests will be performed.  **Analysis of primary endpoint :**  Lowest oxygen saturation measured using MASIMO oximeter, during the interval between the first and the end of the second minute after successful ETI (32) will be compared between groups using linear mixed-effect regression model to take into account stratification variable (centre as random effect, intubator status (expert or non-expert) as fixed effect).  **Sensitivity analysis of primary endpoint**  The same analysis will be realized using the lowest oxygen saturation measured by local oximeter.  **Analysis of primary endpoint according to subgroup**  Primary endpoint will be analyzed according to pre-defined subgroups variables:   - Body mass index (<30 or ≥30) - Reason for ETI: hypoxaemia/other - Shock/No shock at inclusion - Difficult intubation (yes or no) - ORI threshold (<0.6 or ≥0.6)   **Analysis of secondary endpoints**  All analyses will be adjusted on stratification criterion.   - Occurrence of at least one severe life-threatening complication: death, cardiac arrest, arterial systolic pressure <90 mmHg, and/or SpO_2_<80% and occurrence of each one using logistic mixed-effect regression model. - The rates of death in ICU and at 28 days will be calculated via Kaplan-Meier plots and compared between groups using Cox regression models. - ICU and hospital length of stay will be compared using linear mixed-effect regression models. - Cognitive score evaluated by F-TICS-m will be compared between groups using linear mixed effect regression model. Patients dead at day 28 will be not included on this analysis. |

SUMMARY OF PROTOCOL IN FRENCH

| **Titre de l’étude** | **Impact de l’indice de reserve d’oxygène non-invasif par rapport à la norme de soins sur la saturation périphérique en oxygène pendant l’intubation endotrachéale en unite de soins intensifs : essai randomisé de supériorité à 2 bras, multicentrique et ouvert.** |
| --- | --- |
| **Mots clés** | Intubation, hypoxémie, ICU, insuffisance respiratoire aiguë |
| **Sponsor de l’étude** | **CHU de NANTES** |
| **Coordinator**  **(si multicentrique)** | Dr LASCARROU Jean Baptiste  Médecine Intensive Réanimation  CHU de Nantes |
| **Nombre de centres envisagés** | 20 centres en France |
| **Durée de l’étude** | - Durée totale:  13 mois - Période d’inclusion: 12 mois - Période de traitement du participant : 1 heure - Période de suivi du patient : 28 jours |
| **Méthodologie de l’étude** | - Thérapeutique - Multicentrique - Contrôlée, supériorité - Randomisé - Ouvert avec évaluation unifiée des résultats - Prospective |
| **Nombre total de patients inclus** | 950 |
| **Objectifs de l’étude** | Objectif principal : Déterminer si la surveillance de l’ORI augmente le niveau de saturation en oxygène le plus bas pendant l’intervalle entre la première laryngoscopie (définie comme l’introduction du laryngoscope dans la bouche) et la fin de la deuxième minute après la réussite de l’ETI.  Objectifs secondaires :   - Déterminer si la surveillance de l’ORI augmente le niveau de saturation en oxygène le plus bas selon l’oxymètre local - Déteminer si la surveillance de l’ORI augmente le niveau de saturation en oxygène le plus bas en fonction des sous-groupes prédéfini - Évaluer la sécurité de la surveillance de l’ORI - Évaluer l’efficacité de la surveillance de l’ORI |
| **Critères d’évaluation de l’étude** | Critère d’évaluation principal : Saturation en oxygène la plus basse mesurée par l’oxymètre Masimo Rad7 pendant l’intervalle entre la première laryngoscopie (définie comme l’introduction du laryngoscope dans la bouche) et la fin de la deuxième minute après la réussite de l’ETI.  Critère(s) d’évaluation secondaire(s):   - Analyse de la sensibilité du critère d’évaluation principal - Saturation en oxygène la plus basse, mesurée par un oxymètre local, pendant l’intervalle entre la première laryngoscopie - Saturation en oxygène la plus basse dans des sous-groupes prédéfinis - Indice de masse corporelle (<30 ou ≥30) - Motif de l’ETI: hypoxémie/autre - État de choc/absence d’état de choc à l’inclusion - Intubation difficile (oui ou non) - Seuil de l’ORI (<0,6 ou ≥0,6) - Résultat de sécurité : Survenue d’au moins une complication grave mettant en jeu le prognostic vital : décès, arrêt cardiaque, pression artérielle systolique <90mmHg, et/ou Sp02<80% et survenue de chacune d’entre-elles - Résultats d’efficacité - Mortalité en USI - Mortalité à 28 jours en USI à l’hôpital sur durée du séjour - Score cognitif moyen à 28 jours entre les deux groupes (F-TICS-m) |
| **Traitement, procedure interventionnelle à l’étude** | Surveillance de l’ORI pendant la préoxygénation des patients admis en unite de soins intensifs et nécessitant une supplementation en oxygène |
| **Comparateur (si applicable)** | Soins standard (3 minutes de préoxygénation avec un dispositif à la discretion du médecin) |
| **Critères d’inclusion et de non-inclusion** | Critères d’inclusion:   - Admission en unite de soins intensifs et nécessité d’intubation pour permettre une ventilation mécanique - Nécessité d’un apport supplémentaire d’oxygène (par n’importe quell dispositif et à n’importe quel débit) pour obtenir une SpO2>97% - Patient ou proche parent informé de l’étude et ayant consenti à la participation du patient à l’étude (les patients dans le coma sont incapables de donner leur consentement). Si le patient est inapte et qu’aucun proche ne peut-être contacté au moment de l’inclusion dans l’étude, l’inclusion dans l’essai sera réalisée par procédure d’ urgence par le médecin de l’unité de soins intensifs, conformément à la loi française - Patients affiliés à un régime de sécurité sociale   Critères de non-inclusion:   - Intubation par fibroscopie nécessaire selon le médecin responsable - Contre-indications à la laryngoscopie (par exemple, lésion spinale instable) - Délai insuffisant pour inclure et randomiser le patient (par exemple, en raison d’un arrêt cardiaque) - Âge <18 ans - Grossesse ou allaitement - Détenu dans un établissement pénitentaire - Sous tutelle, curatelle ou sous protection de la justice |
| **Calendrier des différentes visites et examens** | \| **Activities** \| J0  (Inclusion visit) \| J1 to J28 \| J28 \| \| --- \| --- \| --- \| --- \| \| Information aux participants \| X \|  \|  \| \| Consentement \| X \|  \|  \| \| Randomisation \| X \|  \|  \| \| Antécédents médicaux \| X \|  \|  \| \| Examens cliniques \| X \| X \|  \| \| Évaluation de l’efficacité et/ou de la sécurité \| X \| X \|  \| \| Examens paracliniques \| X \|  \|  \| \| Compliance \| X \|  \|  \| \| Effets indésirables \|  \| X \| X \| \| Appels téléphoniques \|  \|  \| X \| |
| **Analyses statistiques** | Les analyses statistiques seront réalisées par un biostatisticien de la plateforme méthodologique et biostastitique utilisant SAS ou R ou Stata.  Une revue des données sera réalisée avant le gel des bases de données des essais cliniques et des analyses statistiques, avec les principaux acteurs de l’étude (investigateur, ARC, data manager et biostatistician, …). L’objectif de cette revue sera de faire le point sur les difficultés rencontrées au cours de l’étude, et de classer les différentes deviations afin de définir les populations d’analyse.  **Description des groupes au départ**  Les caractéristiques de base seront étudiées à l’aide de statistiques descriptives. Aucun test statistique ne sera effectué.  **Analyse du critère d’évaluation principal**  La plus faible saturation en oxygène mesurée à l’aide de l’oxymètre MASIMO, pendant l’intervalle entre la première et la fin de la deuxième minute après une ETI réussie (32) sera comparée entre les groupes à l’aide d’un modèle de regression linéaire à effects mixtes pour prendre en compte la variable de stratification (centre comme effet aléatoire, statut de l’intubateur (expert ou non-expert) comme effet fixe).  Analyse de sensibilité du critère d’évaluation principal  La même analyse sera réalisée en utilisant la saturation en oxygène la plus basse mesurée par un oxymètre local.  **Analyse du critère d’évaluation primaire en fonction des sous-groupes**  Le critère d’évaluation primaire sera analysé en fonction des variables des sous-groupes prédéfinis :   - Indice de masse corporelle (<30 ou ≥30) - Motif de l’ETI : hypoxémie/autre - État de choc/absence d’état de choc à l’inclusion - Intubation difficile (oui ou non) - Seuil ORI (<0,6 ou ≥0,6)   **Analyse des critères d’évaluation secondaires**  Toutes les analyses seront ajustées sur le critère de stratification.   - Survenue d’au moins une complication grave menaçant le prognostic vital: décès, arrêt cardiaque, pression artérielle systolique <90mmHg, et/ou SpO2<80% et survenue de chacune d’entre-elles à l’aide d’un modèle de regression logistique à effets mixtes - Les taux de décès en reanimation et à 28 jours seront calculés à l’aide de diagrammes de Kaplan-Meier et comparés entre les groupes à l’aide de modèles de regression linéaire à effets mixtes - Le score cognitive évalué par F-TICS-m sera comparé entre les groupes à l’aide d’un modèle de regression linéaire à effets mixtes. Les patients décédés au 28ème jour ne seront pas inclus dans cette analyse |

Appendix 3: Bibliographic references

1. Russotto V, Myatra SN, Laffey JG, Tassistro E, Antolini L, Bauer P, et al. Intubation Practices and Adverse Peri-intubation Events in Critically Ill Patients From 29 Countries. JAMA. 23 mars 2021;325(12):1164‑72.

2. Jaber S, Amraoui J, Lefrant JY, Arich C, Cohendy R, Landreau L, et al. Clinical practice and risk factors for immediate complications of endotracheal intubation in the intensive care unit: a prospective, multiple-center study. Crit Care Med. sept 2006;34(9):2355‑61.

3. De Jong A, Rolle A, Molinari N, Paugam-Burtz C, Constantin JM, Lefrant JY, et al. Cardiac Arrest and Mortality Related to Intubation Procedure in Critically Ill Adult Patients: A Multicenter Cohort Study. Crit Care Med. avr 2018;46(4):532‑9.

4. Lascarrou JB, Boisrame-Helms J, Bailly A, Le Thuaut A, Kamel T, Mercier E, et al. Video Laryngoscopy vs Direct Laryngoscopy on Successful First-Pass Orotracheal Intubation Among ICU Patients: A Randomized Clinical Trial. Jama. 7 févr 2017;317(5):483‑93.

5. McKown AC, Casey JD, Russell DW, Joffe AM, Janz DR, Rice TW, et al. Risk Factors for and Prediction of Hypoxemia during Tracheal Intubation of Critically Ill Adults. Ann Am Thorac Soc. 2018;15(11):1320‑7.

6. Mort TC. The incidence and risk factors for cardiac arrest during emergency tracheal intubation: a justification for incorporating the ASA Guidelines in the remote location. J Clin Anesth. nov 2004;16(7):508‑16.

7. Higgs A, McGrath BA, Goddard C, Rangasami J, Suntharalingam G, Gale R, et al. Guidelines for the management of tracheal intubation in critically ill adults. Br J Anaesth. févr 2018;120(2):323‑52.

8. Quintard H, l’Her E, Pottecher J, Adnet F, Constantin JM, De Jong A, et al. Experts’ guidelines of intubation and extubation of the ICU patient of French Society of Anaesthesia and Intensive Care Medicine (SFAR) and French-speaking Intensive Care Society (SRLF) : In collaboration with the pediatric Association of French-speaking Anaesthetists and Intensivists (ADARPEF), French-speaking Group of Intensive Care and Paediatric emergencies (GFRUP) and Intensive Care physiotherapy society (SKR). Ann Intensive Care. 22 janv 2019;9(1):13.

9. Mosier JM, Hypes CD, Sakles JC. Understanding preoxygenation and apneic oxygenation during intubation in the critically ill. Intensive Care Med. févr 2017;43(2):226‑8.

10. Menk M, Estenssoro E, Sahetya SK, Neto AS, Sinha P, Slutsky AS, et al. Current and evolving standards of care for patients with ARDS. Intensive Care Med. 1 déc 2020;46(12):2157‑67.

11. Frat JP, Ricard JD, Coudroy R, Robert R, Ragot S, Thille AW. Preoxygenation with non-invasive ventilation versus high-flow nasal cannula oxygen therapy for intubation of patients with acute hypoxaemic respiratory failure in ICU: the prospective randomised controlled FLORALI-2 study protocol. BMJ Open. 2017;7(12).

12. Frat JP, Ricard JD, Quenot JP, Pichon N, Demoule A, Forel JM, et al. Non-invasive ventilation versus high-flow nasal cannula oxygen therapy with apnoeic oxygenation for preoxygenation before intubation of patients with acute hypoxaemic respiratory failure: a randomised, multicentre, open-label trial. Lancet Respir Med. avr 2019;7(4):303‑12.

13. Baillard C, Fosse JP, Sebbane M, Chanques G, Vincent F, Courouble P, et al. Noninvasive ventilation improves preoxygenation before intubation of hypoxic patients. Am J Respir Crit Care Med. 15 juill 2006;174(2):171‑7.

14. Baillard C, Prat G, Jung B, Futier E, Lefrant JY, Vincent F, et al. Effect of preoxygenation using non-invasive ventilation before intubation on subsequent organ failures in hypoxaemic patients: a randomised clinical trial. Br J Anaesth. févr 2018;120(2):361‑7.

15. Janz DR, Casey JD, Semler MW, Russell DW, Dargin J, Vonderhaar DJ, et al. Effect of a fluid bolus on cardiovascular collapse among critically ill adults undergoing tracheal intubation (PrePARE): a randomised controlled trial. Lancet Respir Med. déc 2019;7(12):1039‑47.

16. Mort TC, Waberski BH, Clive J. Extending the preoxygenation period from 4 to 8 mins in critically ill patients undergoing emergency intubation. Crit Care Med. janv 2009;37(1):68‑71.

17. Scheeren TWL, Belda FJ, Perel A. The oxygen reserve index (ORI): a new tool to monitor oxygen therapy. J Clin Monit Comput. juin 2018;32(3):379‑89.

18. Yoshida K, Isosu T, Noji Y, Hasegawa M, Iseki Y, Oishi R, et al. Usefulness of oxygen reserve index (ORi^TM^), a new parameter of oxygenation reserve potential, for rapid sequence induction of general anesthesia. J Clin Monit Comput. août 2018;32(4):687‑91.

19. Szmuk P, Steiner JW, Olomu PN, Ploski RP, Sessler DI, Ezri T. Oxygen Reserve Index: A Novel Noninvasive Measure of Oxygen Reserve--A Pilot Study. Anesthesiology. avr 2016;124(4):779‑84.

20. Hille H, Le Thuaut A, Canet E, Lemarie J, Crosby L, Ottavy G, et al. Oxygen reserve index for non-invasive early hypoxemia detection during endotracheal intubation in intensive care: the prospective observational NESOI study. Ann Intensive Care. 17 juill 2021;11(1):112.

21. Martin M, Decamps P, Seguin A, Garret C, Crosby L, Zambon O, et al. Nationwide survey on training and device utilization during tracheal intubation in French intensive care units. Ann Intensive Care. 3 janv 2020;10(1):2.

22. Casey JD, Janz DR, Russell DW, Vonderhaar DJ, Joffe AM, Dischert KM, et al. Bag-Mask Ventilation during Tracheal Intubation of Critically Ill Adults. N Engl J Med. 28 févr 2019;380(9):811‑21.

23. Vercambre MN, Cuvelier H, Gayon YA, Hardy-Léger I, Berr C, Trivalle C, et al. Validation study of a French version of the modified telephone interview for cognitive status (F-TICS-m) in elderly women. Int J Geriatr Psychiatry. nov 2010;25(11):1142‑9.

24. Simpson GD, Ross MJ, McKeown DW, Ray DC. Tracheal intubation in the critically ill: a multi-centre national study of practice and complications. Br J Anaesth. mai 2012;108(5):792‑9.

25. De Jong A, Molinari N, Terzi N, Mongardon N, Arnal JM, Guitton C, et al. Early identification of patients at risk for difficult intubation in the intensive care unit: development and validation of the MACOCHA score in a multicenter cohort study. Am J Respir Crit Care Med. 15 avr 2013;187(8):832‑9.

26. McCrory JW, Matthews JN. Comparison of four methods of preoxygenation. Br J Anaesth. mai 1990;64(5):571‑6.

27. Hett DA, Geraghty IF, Radford R, House JR. Routine pre-oxygenation using a Hudson mask. A comparison with a conventional pre-oxygenation technique. Anaesthesia. févr 1994;49(2):157‑9.

28. Driver BE, Prekker ME, Kornas RL, Cales EK, Reardon RF. Flush Rate Oxygen for Emergency Airway Preoxygenation. Ann Emerg Med. 1 janv 2017;69(1):1‑6.

29. Robinson A, Ercole A. Evaluation of the self-inflating bag-valve-mask and non-rebreather mask as preoxygenation devices in volunteers. BMJ Open. 1 janv 2012;2(5).

30. Guitton C, Ehrmann S, Volteau C, Colin G, Maamar A, Jean-Michel V, et al. Nasal high-flow preoxygenation for endotracheal intubation in the critically ill patient: a randomized clinical trial. Intensive Care Med. 2019;45(4):447‑58.

31. Vourc’h M, Asfar P, Volteau C, Bachoumas K, Clavieras N, Egreteau PY, et al. High-flow nasal cannula oxygen during endotracheal intubation in hypoxemic patients: a randomized controlled clinical trial. Intensive Care Med. sept 2015;41(9):1538‑48.

32. Driver BE, Semler MW, Self WH, Ginde AA, Trent SA, Gandotra S, et al. Effect of Use of a Bougie vs Endotracheal Tube With Stylet on Successful Intubation on the First Attempt Among Critically Ill Patients Undergoing Tracheal Intubation: A Randomized Clinical Trial. JAMA. 28 déc 2021;326(24):2488‑97.

33. Jaber S, Rollé A, Godet T, Terzi N, Riu B, Asfar P, et al. Effect of the use of an endotracheal tube and stylet versus an endotracheal tube alone on first-attempt intubation success: a multicentre, randomised clinical trial in 999 patients. Intensive Care Med. juin 2021;47(6):653‑64.

34. Bailly A, Ricard JD, Le Thuaut A, Helms J, Kamel T, Mercier E, et al. Compared Efficacy of Four Preoxygenation Methods for Intubation in the ICU: Retrospective Analysis of McGrath Mac Videolaryngoscope Versus Macintosh Laryngoscope (MACMAN) Trial Data. Crit Care Med. 30 janv 2019;

35. Decamps P, Grillot N, Le Thuaut A, Brule N, Lejus-Bourdeau C, Reignier J, et al. Comparison of four channelled videolaryngoscopes to Macintosh laryngoscope for simulated intubation of critically ill patients: the randomized MACMAN2 trial. Ann Intensive Care. 16 août 2021;11(1):126.

Appendix 4: Emergency situation attestation form

APPENDIX 5 : Information Patient form

APPENDIX 5bis: Relative consent form

APPENDIX 6: RELATIVE INFORMATION FORM

APPENDIX 6BIS: Relative Consent Form

APPENDIX 7: Information Patient form, after emergency situation

APPENDIX 7bis: Patient CONSENT form, after emergency situation

Appendix 8: Relative information form, after emergency situation

Appendix 8bis: Relative consent form, after emergency situation
