# Supplementary material for: Impact of non-invasive oxygen reserve index versus standard SpO2 monitoring on peripheral oxygen saturation during endotracheal intubation in the intensive care unit: Protocol for the randomized controlled trial NESOI2
Source: PLoS One. 2024 Sep 16;19(9):e0307723. doi: 10.1371/journal.pone.0307723 (PMC11404791; doi:10.1371/journal.pone.0307723)
Supplement: Attachment — Submitted filename: 2024_ORI_ICU_PlosOne.docx [file pone.0307723.s003.docx]

Impact of non-invasive oxygen reserve index versus standard SpO2 monitoring on peripheral oxygen saturation during endotracheal intubation in the intensive care unit: Protocol for the randomized controlled trial NESOI

Thank you for considering me for this review.

This is a protocol for a multicenter, two-arm, 83 parallel-group, randomized, superiority, open trial in 950 critically ill adults requiring endo tracheal intubation (ETI) aiming at monitoring ORI values and using an oxygen reserve index (ORI) target for 85 preoxygenation of at least 0.6 for at least 1 minute as compared to a standard of care SpO2 monitoring.

The protocol is well written and could have significant implications on the care of this vulnerable patient group by potentially preventing desaturations.

Here are my comments:

- ICU patients needing intubation are a heterogenous population. I think including a respiratory failure patient or a congestive cardiac patient with a trauma or septic patient would skew the results. The study would benefit from a more homogenous study population.
- Since ORI values represent a PaO2 of >100mmHg none of the patients with SpO2 <98%, will have a measurable ORI value. Therefore, I would suggest explicitly excluding patients who do not reach an SpO2 of 99-100% after 3-5 min of preoxygenation.
- P 9, first paragraph – the primary outcome measurement time (lowest SpO2 value, first introduction of the laryngoscope into the mouth and the end of the second 189 minute following successful intubation) seems too short to me. Two minutes after intubation could be too short for SpO2 to recover after apnea in critically ill patients.
- P 9, R 197 – As mentioned above, ORI will be 0 at SpO2 >98%, thus it can’t assess severe hypoxic events
