# Supplementary material for: Impact of non-invasive oxygen reserve index versus standard SpO2 monitoring on peripheral oxygen saturation during endotracheal intubation in the intensive care unit: Protocol for the randomized controlled trial NESOI2
Source: PLoS One. 2024 Sep 16;19(9):e0307723. doi: 10.1371/journal.pone.0307723 (PMC11404791; doi:10.1371/journal.pone.0307723)
Supplement: Attachment — Submitted filename: Editor and Reviewer Answer(aw).docx [file pone.0307723.s004.docx]

1. Please ensure that your manuscript meets PLOS ONE's style requirements, including those for file naming. The PLOS ONE style templates can be found at

<https://journals.plos.org/plosone/s/file?id=wjVg/PLOSOne_formatting_sample_main_body.pdf> <https://journals.plos.org/plosone/s/file?id=ba62/PLOSOne_formatting_sample_title_authors_affiliations.pdf>

**Reply 1.** We have checked compliance of the manuscript with *PLOS ONE* style requirements.

2. We note that the grant information you provided in the ‘Funding Information’ and ‘Financial Disclosure’ sections do not match.

When you resubmit, please ensure that you provide the correct grant numbers for the awards you received for your study in the ‘Funding Information’ section.

**Reply 2.** We apologize for this error, which has been corrected. The correct grant number is PHRC-21-0202.

3. Thank you for stating the following financial disclosure:

“French Ministry of Health. PHRC 2021.”

Please state what role the funders took in the study.  If the funders had no role, please state: “The funders had no role in study design, data collection and analysis, decision to publish, or preparation of the manuscript.”

Please include this amended Role of Funder statement in your cover letter; we will change the online submission form on your behalf.

**Reply 3.** The manuscript now contains the following statement: “The funder had no role in designing the trial and will have no role in collecting, analyzing, or interpreting the data; writing the trial report; or deciding to submit the trial report for publication. The funder also had no role in writing the present article or deciding to submit it for publication.”

We have added the role-of-funder statement to the cover letter.

4. Thank you for stating the following in the Competing Interests section:

“JBL has received lecturing fees from BD and Masimo. Alexis Ferré reports honoraria by Fisher & Paykel for a lecture during SFMU Congress 2022, outside the submitted work. All other authors reports not competing interests.”

Please confirm that this does not alter your adherence to all PLOS ONE policies on sharing data and materials, by including the following statement: "This does not alter our adherence to  PLOS ONE policies on sharing data and materials.” (as detailed online in our guide for authors http://journals.plos.org/plosone/s/competing-interests).  If there are restrictions on sharing of data and/or materials, please state these. Please note that we cannot proceed with consideration of your article until this information has been declared.

Please include your updated Competing Interests statement in your cover letter; we will change the online submission form on your behalf.

**Reply 4.** We confirm that we will adhere to all *PLOS ONE* policies on sharing data and materials. This information has been added to the Competing Interest statement in the manuscript and cover letter.

5. One of the noted authors is a group or consortium [CRICS-TRIGGERSEP Network]. In addition to naming the author group, please list the individual authors and affiliations within this group in the acknowledgments section of your manuscript. Please also indicate clearly a lead author for this group along with a contact email address.

**Reply 5.** The members of the CRICS-TRIGGERSEP Network are listed in the Acknowledgments section.

6. We note that Figure 4 in your submission contain copyrighted images. All PLOS content is published under the Creative Commons Attribution License (CC BY 4.0), which means that the manuscript, images, and Supporting Information files will be freely available online, and any third party is permitted to access, download, copy, distribute, and use these materials in any way, even commercially, with proper attribution. For more information, see our copyright guidelines: http://journals.plos.org/plosone/s/licenses-and-copyright. We require you to either (1) present written permission from the copyright holder to publish these figures specifically under the CC BY 4.0 license, or (2) remove the figures from your submission:

1. You may seek permission from the original copyright holder of Figure 4 to publish the content specifically under the CC BY 4.0 license.

We recommend that you contact the original copyright holder with the Content Permission Form (http://journals.plos.org/plosone/s/file?id=7c09/content-permission-form.pdf) and the following text:

“I request permission for the open-access journal PLOS ONE to publish XXX under the Creative Commons Attribution License (CCAL) CC BY 4.0 (http://creativecommons.org/licenses/by/4.0/). Please be aware that this license allows unrestricted use and distribution, even commercially, by third parties. Please reply and provide explicit written permission to publish XXX under a CC BY license and complete the attached form.”

Please upload the completed Content Permission Form or other proof of granted permissions as an “Other” file with your submission.

In the figure caption of the copyrighted figure, please include the following text: “Reprinted from [ref] under a CC BY license, with permission from [name of publisher], original copyright [original copyright year].”

2. If you are unable to obtain permission from the original copyright holder to publish these figures under the CC BY 4.0 license or if the copyright holder’s requirements are incompatible with the CC BY 4.0 license, please either i) remove the figure or ii) supply a replacement figure that complies with the CC BY 4.0 license. Please check copyright information on all replacement figures and update the figure caption with source information. If applicable, please specify in the figure caption text when a figure is similar but not identical to the original image and is therefore for illustrative purposes only.

**Reply 6.** Please find approval in eAppendix.

7. We note that the original protocol that you have uploaded as a Supporting Information file contains an institutional logo. As this logo is likely copyrighted, we ask that you please remove it from this file and upload an updated version upon resubmission.

**Reply 7.** We have removed the logo.

Additional Editor Comments:

Thank you for submitting the protocol of interesting study.

**Reply 8.** We thank the editor for this positive comment.

Please see reviewer's comment.

**Reviewer #1:** Thank you for sharing the manuscript titled "Impact of Non-Invasive Oxygen Reserve Index Versus Standard SpO2 Monitoring on Peripheral Oxygen Saturation During Endotracheal Intubation in the Intensive Care Unit: Protocol for the Randomized Controlled Trial NESOI2" for review.

The paper outlines a research protocol assessing the impact of preoxygenation guided by the Oxygen Reserve Index (ORI) on the severity of desaturation during orotracheal intubation in ICU settings.

The manuscript is well-written and largely unambiguous. The methodology is thoroughly described and detailed. I have no significant comments.

We are encouraged by this positive evaluation.

Abstract

1. Line 85: “ORI target for preoxygenation of at least 0.6 for at least 1 minute.” Please explain the rational“ for selecting this specific target.

**Reply 1.** As indicated in the “Intervention arm” section, the 0.6 cutoff was chosen based on unpublished data from the NESOI1 study (Hille et al., Annals of Intensive Care, 2020). The 0.6 value was consistent with the expected high PaO_2_ value and with the proportion of patients expected to achieve this value during preoxygenation.

2. Line 85: “Preoxygenation is guided by SpO2 values recorded by a standard pulse oximeter, according to the standard of care.” Could you describe how SpO2 guides preoxygenation? (Is it aiming for a SpO2 of 100% for a specific duration?).

**Reply 2.** We agree with the Reviewer that this point requires clarification, given the absence of a preoxygenation SpO_2_ target in the literature. We have added that the goal was to obtain an SpO_2_ of 100% during preoxygenation, which lasted at least 3 minutes.

Main Manuscript

3. Line 189: Please explain the relevance of reference 19 in the methodology. Does it describe a study with a similar methodology? Or is it related to another aspect? In any case, if the reference is cited to justify methodological choices (such as the rationale behind the decision to monitor saturation 2 minutes post-intubation), it would be more appropriate to include it in the discussion section.

**Reply 3.** Reference 19 is given to support the timing of primary outcome evaluation. We have transferred it to the discussion section as suggested by the Reviewer.

4. Line 190: “The secondary outcome includes the sensitivity analysis of the primary outcome.” This analysis pertains to alternative statistical presentations of the primary endpoint. I suggest relocating this alongside the calculations for the primary endpoint.

**Reply 4.** This sentence now immediately follows the description of the analysis of the primary outcome, with the other secondary outcomes in the next paragraph.

5. Line 257: "Chosen based on unpublished personal data (JBL)." Please provide a brief description of what these data have shown.

**Reply 5.** An ORI of 0.6 corresponds to a PaO_2_ of 160 mmHg (Applegate et al., Anaesth Analg 2016). In the NESOI1 trial, the median ORI value at the end of preoxygenation was 0.62 [0.26–0.83]. In NESOI1 the median ORI in patients whose SpO_2_ did not fall below 90% during intubation was 0.68 [0.27–0.89). In NESOI1, the highest median ORI during preoxygenation was 0.77 [0.59–1.00] in patients with desaturation defined as SpO_2_<97% vs. 0.49 [0.22–0.77] in patients with desaturation.

Thus, an ORI cutoff of 0.6 is a good compromise given the expected hyperoxia, theoretical maximum PaO_2_ according to Masimo (the ORI device manufacturer), and proportion of patients expected to achieve ORI≥0.6 in a large clinical trial.

6. Line 264: “The intensivist will not be aware of the SpO2 and ORI values”…" If the intensivist is unaware of these monitors, on what basis will he determine the duration of preoxygenation?

**Reply 6.** The full sentence is “The intubating intensivist will not be aware of the SpO_2_ and ORI values provided by the Rad-97 monitor throughout the ETI procedure, which will be recorded by the clinical research nurse.” The intubating intensivist is aware of the SpO_2_ values recorded by the standard pulse oximeter.

7. Line 291: “Intubating intensivist...” If the choice of drugs is left to the discretion of the intensivist, I see no value in citing recommendations.

**Reply 7.** The protocol highlights the existence of recommendations, which can guide the intensivists in their choices. We agree that the original sentence was confusing. We have rewritten it as follows (page 11, lines 267–270): “The intubating intensivists are encouraged to follow international [7] and French [8] recommendations that etomidate (0.2–0.3 mg/kg) and ketamine (1–2 mg/kg) be used as hypnotics, succinylcholine (1 mg/kg) as the first-line muscle relaxant, and rocuronium (1 mg/kg) as the alternative muscle relaxant.”

8. Line 322: The various phases, durations, and definitions could be graphically represented on a timeline, enhancing understanding.

**Reply 8.** We agree with the Reviewer that clarification was needed. A timeline has been added, in the new Figure 5.

9. The choice of alternative devices for initial preoxygenation should be controlled. Suppose the distribution of alternatives to preoxygenation differs between groups. In that case, it might raise questions about whether the difference in preoxygenation quality is related to the monitoring or the preoxygenation tool.

**Reply 9.** We agree with the Reviewer that the safety and efficacy of preoxygenation depend in part on the device used and that all devices may not be available in all participating ICUs. However, the intubating intensivists are free to change to a different preoxygenation device should oxygenation remain below the target so preoxygenation devices are directly linked with the intervention.

**Reviewer #2:** Impact of non-invasive oxygen reserve index versus standard SpO2 monitoring on peripheral oxygen saturation during endotracheal intubation in the intensive care unit: Protocol for the randomized controlled trial NESOI. Thank you for considering me for this review. This is a protocol for a multicenter, two-arm, 83 parallel-group, randomized, superiority, open trial in 950 critically ill adults requiring endo tracheal intubation (ETI) aiming at monitoring ORI values and using an oxygen reserve index (ORI) target for 85 preoxygenation of at least 0.6 for at least 1 minute as compared to a standard of care SpO2 monitoring. The protocol is well written and could have significant implications on the care of this vulnerable patient group by potentially preventing desaturations.
We thank the Reviewer for these positive comments.

Here are my comments:
1. ICU patients needing intubation are a heterogenous population. I think including a respiratory failure patient or a congestive cardiac patient with a trauma or septic patient would skew the results. The study would benefit from a more homogenous study population.

**Reply 1.** We agree with the Reviewer that data from homogeneous populations are easier to interpret. However, for this pragmatic trial, our goal was to reflect daily intubation practice. Intubation is performed as a common procedure for patients with a wide spectrum of conditions. Moreover, time to the primary outcome is very short and therefore captures events related to intubation as opposed to the underlying condition. Finally, our use of broad inclusion criteria will improve the external validity of our findings.

2. Since ORI values represent a PaO2 of >100mmHg none of the patients with SpO2 <98%, will have a measurable ORI value. Therefore, I would suggest explicitly excluding patients who do not reach an SpO2 of 99-100% after 3-5 min of preoxygenation.

**Reply 2.** We agree with the Reviewer that patients with SpO_2_<98% will not have ORI values to help guide preoxygenation, in the intervention group. However, with the increasing use of non-invasive ventilation, SpO_2_ values of less than 98% at the end of preoxygenation are very rare. Post-randomization exclusion of patients with SpO_2_<98% at the end of preoxygenation would constitute a methodological weakness.

3. P 9, first paragraph – the primary outcome measurement time (lowest SpO2 value, first introduction of the laryngoscope into the mouth and the end of the second 189 minute following successful intubation) seems too short to me. Two minutes after intubation could be too short for SpO2 to recover after apnea in critically ill patients.

**Reply 3.** We agree with the Reviewer that oxygenation recovery after apnea may require more than 2 minutes. However, we chose this short interval to avoid a potential influence of ventilator setting modifications to manage hypoxemia and to ensure that the primary outcome is related to the intubation as opposed to the underlying condition. This choice was made after careful discussion with Prof. Jonathan Casey from the Pragmatic Critical Care Trial Group (please also see the reply regarding the PREVENT trial in the *New England Journal of Medicine*). We have added the following at the end of the discussion section (page 17, lines 406–409): “The primary outcome (lowest SpO_2_) is recorded from the beginning of laryngoscopy to the end of the second minute after successful intubation. This duration was chosen to avoid interactions with ventilator setting changes, in accordance with a previous study.”

4. P 9, R 197 – As mentioned above, ORI will be 0 at SpO2 >98%, thus it can’t assess severe hypoxic events

**Reply 4.** We agree with the Reviewer that the ORI values will not detect severe hypoxemia. However, in all trial patients, the SpO_2_ value will be available. ORI is used in the trial to guide over-oxygenation before intubation, not to detect hypoxemia. Of note, an ORI drop has been reported to provide early warning about impending hypoxemia, before the SpO_2_ drop (Scheeren et al., J Clin Monit Comput, 2018; ).
