# Supplementary material for: Impact of non-invasive oxygen reserve index versus standard SpO2 monitoring on peripheral oxygen saturation during endotracheal intubation in the intensive care unit: Protocol for the randomized controlled trial NESOI2
Source: PLoS One. 2024 Sep 16;19(9):e0307723. doi: 10.1371/journal.pone.0307723 (PMC11404791; doi:10.1371/journal.pone.0307723)
Supplement: Attachment — Submitted filename: Reviewer Answer R2(aw).docx [file pone.0307723.s005.docx]

**Reviewer #1:** Thank you for your revisions and for addressing the initial comments on your manuscript. Upon further review, I have two additional suggestions that could strengthen your submission:

Responses to comments 1, 5, and 9 from Reviewer #1 should be incorporated into the discussion section of the article to better justify and explain the methodological choices made.

As requested by the Reviewer, we have added the reasons for and explanations of our methodological choices to the discussion section.

Concerning Reviewer #2’s Comments: I understand that the authors prefer not to exclude patients with SpO₂ below 98% at the end of preoxygenation due to their rarity. However, it is essential to clearly describe in the statistical section how their data will be analyzed should this issue arise.

Patients with SpO_2_ below 98% at the end of preoxygenation will be included in the modified intention-to-treat population, on which the main analysis will be performed, as now stated in the statistical section of the manuscript.

**Reviewer #2:** The responses to my initial review have been answered. I accept the author's comments and I agree with the publication

We thank the Reviewer for agreeing with publication of our protocol.
