# Supplementary material for: Impact of non-invasive oxygen reserve index versus standard SpO2 monitoring on peripheral oxygen saturation during endotracheal intubation in the intensive care unit: Protocol for the randomized controlled trial NESOI2
Source: PLoS One. 2024 Sep 16;19(9):e0307723. doi: 10.1371/journal.pone.0307723 (PMC11404791; doi:10.1371/journal.pone.0307723)
Supplement: Attachment — Submitted filename: Reviewer Answer R3(aw).docx [file pone.0307723.s006.docx]

Reviewer #1: I thank the authors for adequately addressing the reviewers' comments. I have no further comments to add.

We thank the Reviewer for agreeing with publication of our protocol.

Reviewer #3: On the overall, the manuscript addresses previously raised questions. My additional comments are as follows:

1. A linear mixed model will be fitted, which is based on Gaussian assumptions of the random terms (random effects & errors). What would be the recourse, when the Gaussian assumptions fail (which can certainly happen)?

We add details in the manuscript about Statistical Analysis Plan (SAP) if Gaussian assumptions fail.

2. Cox regression will be employed, but necessary goodness of fit statistics and tests of the proportional hazards assumptions need to be mentioned.

We add details in the manuscript about Statistical Analysis Plan (SAP) if proportional hazards assumptions fail.

We hope you will now find our work suitable for publication.
